# Self-Instruction Video Versus Face-to-Face Instruction of Pharmacy Students’ Skills in Blood Pressure Measurement

**DOI:** 10.3390/pharmacy8040217

**Published:** 2020-11-16

**Authors:** Samieh Farahani, Imaneh Farahani, Bjoern B. Burckhardt, Holger Schwender, Stephanie Laeer

**Affiliations:** 1 Institute of Clinical Pharmacy and Pharmacotherapy, Heinrich Heine University Duesseldorf, Universitaetsstrasse 1, 40225 Duesseldorf, Germany; imaneh.farahani@hhu.de (I.F.); bjoern.burckhardt@hhu.de (B.B.B.); stephanie.laeer@hhu.de (S.L.); 2 Mathematical Institute, Heinrich Heine University Duesseldorf, Universitaetsstrasse 1, 40225 Duesseldorf, Germany; holger.schwender@hhu.de

**Keywords:** pharmacy education, pharmacy students, blood pressure measurement, educational video, self-instructional video

## Abstract

A modern approach to clinical skill education is the use of educational videos, yet there is a shortage of literature investigating the effect of self-instruction videos (SIVs) in pharmacy students. Therefore, our objective was to investigate whether an SIV is non-inferior compared to face-to-face instruction (FTFI) in acquiring blood pressure measurement skills. The participants in this randomized controlled study were pharmacy students. The control group was taught by FTFI, while the intervention group watched an SIV. Before and after the instruction, the participants’ performance was assessed by an objective structured clinical examination (OSCE). The participants completed a self-assessment survey before each OSCE session. Moreover, the participants’ perception and satisfaction were assessed using another survey. The OSCE score and self-assessment score increased significantly from pre- to post-instruction in both groups. The SIV was non-inferior compared to FTFI in terms of the OSCE score, considering a predefined non-inferiority margin of −10%. The participants’ self-assessment yielded inconclusive results for non-inferiority. Both instructional approaches were well received. Considering our findings, SIVs might be a valuable option for teaching pharmacy students’ blood pressure measurement skills. However, depending on the skill intended to be taught, a combination of an instructional video with instructor-led teaching may be necessary.

## 1. Introduction

High blood pressure (BP) is the leading risk factor of death from cardiovascular diseases, chronic kidney disease, and diabetes in every region, causing over 40% of worldwide deaths from these diseases in 2010 [1]. In the same year, the estimated worldwide number of adults with hypertension was 1.39 billion. An estimated 46.5% of adults with hypertension were aware of their disease, while 36.9% were treated with antihypertensive medication. However, BP was controlled in only 13.8% of adults with hypertension [2]. Early detection, treatment, and control of hypertension are crucial for decreasing hypertension-associated morbidity and mortality [2,3,4].

Pharmacists are well positioned to support the management of hypertension. They are not only one of the most accessible health professionals [5,6], but can also perform multifaceted activities such as patient health education, BP measurements, teaching patients about BP self-measurement, patient counseling and information on drug treatment, medication management, and medication reminders [7,8]. In 2018, the updated European Society of Cardiology/European Society of Hypertension (ESC/ESH) Guidelines for the management of arterial hypertension emphasized “a key role for nurses and pharmacists in the longer-term management of hypertension” [9]. The literature demonstrates the beneficial effect of a pharmacist’s intervention alone or in collaboration with other healthcare professionals in improving BP control [10,11,12,13].

Although BP measurement is a common procedure, various factors can affect the accuracy of BP measurement, which should be considered by healthcare providers [14]. Inaccurate BP measurement, often caused by improper procedures during BP measurement, bears the risk of misclassification and subsequent inadequate pharmacotherapy [15,16]. As the “accuracy of BP readings relies on standardized techniques and appropriate observer training” [17], this competence should be taught at an early stage, namely during pharmacy studies.

Traditional teaching approaches, such as face-to-face teaching, have been used for clinical skill education for decades. However, it may be influenced by the instructors’ ability to ensure that all students are engaged in learning [18]. The quality and content of face-to-face teaching tends to vary between different instructors [19]. Modern approaches, such as educational videos, might be a promising alternative. In a face-to-face environment, students have the opportunity to interact with the lecturer during the lecture [20], whereas the advantage of educational videos is that learners can be taught in a standardized manner, which contributes to the reduction of procedural and methodological inconsistencies in skills teaching [19,21,22,23]. The advantage of making an educational video available online is that it can reach many geographically dispersed learners [24]. Particularly in the current COVID-19 pandemic, when many educational institutions have been temporarily closed [25,26], the vital role of educational videos as a tool for distance education has become apparent. The literature describes several types of educational videos, such as video lectures [27], clinical skill demonstration videos [28], and video cases [29]. Instructional videos are studied in addition to traditional instructor-led teaching [30,31], or as a replacement for traditional instructor-led teaching as self-directed learning [18,32,33].

However, there is a shortage of literature investigating the effect of self-instruction videos in pharmacy students compared to a control group. Therefore, the primary objective of this study was to investigate whether a self-instruction video (SIV) is non-inferior to face-to-face instruction (FTFI) in terms of acquiring BP measurement skills. The secondary objectives were to determine whether the SIV is non-inferior in terms of participants’ self-assessment and to explore the participants’ perception and satisfaction.

## 2. Materials and Methods

### 2.1. Operational Definitions

For the purpose of this article, we used the terms:

- Self-instruction video (SIV) to refer to a video recording that demonstrates and explains a skill and that aims to teach the skill without any facilitator or instructor present.
- Face-to-face instruction (FTFI) to refer to an instruction performed by an instructor, whereby the learner and instructor are in the same location and participate in the instruction at the same time [34].

### 2.2. Participants and Study Design

**Figure 1:**
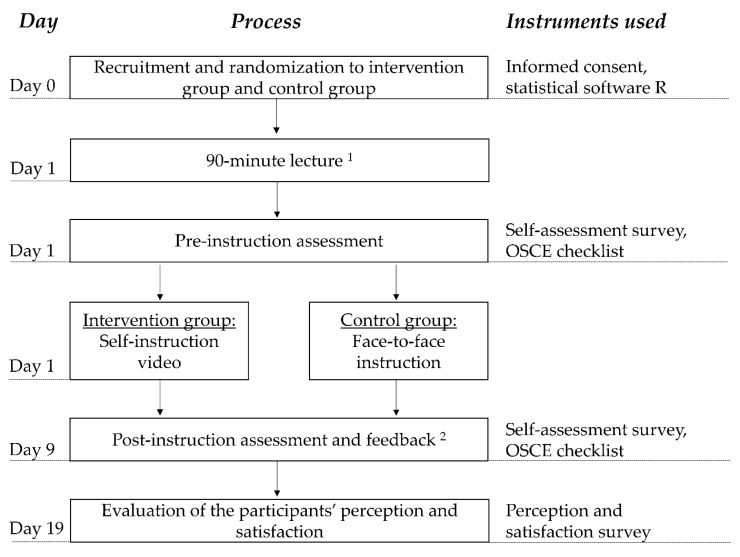
Overview of study design. OSCE = objective structured clinical examination. ^1^ The lecture dealt with the basic knowledge of hypertension and vital parameters, without describing the actual procedure of the oscillometric blood pressure measurement. ^2^ Immediately after the post-instruction OSCE encounter, each participant received feedback from the rater.

Approval for this study was granted by the responsible Ethics Commission (Number 2018-164_2-ProspDEuA). The participants were pharmacy students in the last semester of their university studies at Heinrich Heine University Duesseldorf, Duesseldorf, Germany. The students were included in the study when they had granted voluntary, written informed consent. The investigation was conducted as a randomized controlled non-inferiority trial using a pre-post design. We evaluated the effectiveness of an SIV (intervention group) as an alternative to FTFI (control group). Figure 1 illustrates the study design.

### 2.3. Objective Structured Clinical Examination (OSCE)

Clinical skill performance was measured by pre- and post-instruction OSCEs. Both the pre- and post-instruction OSCEs comprised one station that was limited to 10 min. In both the pre- and post-instruction OSCEs, the participants were required to pretend they were in a community pharmacy and measure the BP of an adult with an oscillometric upper-arm BP monitor (OMRON M5 Professional HEM-7001-D). In the OSCEs, one BP monitor, one measuring tape, three different cuff sizes, and writing utensils were provided. In each OSCE encounter, one participant took over the role of the pharmacist, and in addition, one standardized patient, and one rater were involved. The standardized patients were played by faculty members or volunteer pharmacy students who did not participate in the study. Moreover, the standardized patients had been prepared for their role. In total, four raters supported the study. The rater assessed the participant’s OSCE performance by filling in the OSCE checklist. Each participant was rated by the same rater in the pre- and post-instruction OSCE. The role of the raters was performed by two previously trained pharmacists of the clinical pharmacy faculty and two pharmacy students who had been trained beforehand in the scope of their elective course in their last year of pharmacy studies.

### 2.4. Instruction

#### 2.4.1. Self-Instruction Video

The SIV was developed by two pharmacists with the support of the university’s multimedia center. It depicted the stepwise process of BP measurement with the same upper-arm BP monitor applied in the OSCEs and was structured with the following sections: “intro,” “greeting,” “facilities,” “preparation of blood pressure measurement,” “rest period,” “process of blood pressure measurement,“ “documentation of the blood pressure measurement,” and “final credits”. To improve the viewer’s understanding, the SIV was amended by slides that included pictures and written explanations. The video was in the local language German. Each participant watched the 11 min and 11 s video on an individual computer with headphones in one room at the university.The participants were allowed to watch it for up to approximately 15 min. In this timeframe, the participants could pause, rewind, and replay the video according to their preferences. The video was temporarily available for the SIV group on the computers in the university’s computer room. The teaching was done without the input of an instructor and was self-directed. The participants were instructed not to make notes or other records such as video or audio recordings of the SIV. Typically, taking notes during teaching activities appears to be a regular part of students’ behavior. However, in the scope of the study, the participants were instructed to refrain from that to avoid information exchange between the groups and potentially correlated confounders.

#### 2.4.2. Face-to-Face Instruction

The participants in the FTFI group received FTFI for approximately 15–20 min by an instructor. The role of the instructors was performed by three faculty members (pharmacists). Students, who did not sign the informed consent form, attended the FTFI, leading to an instructor:participant ratio of 1:11. In the FTFI, the process of BP measurement was explained and demonstrated. The participants of the FTFI group were also instructed not to make notes of the instruction for the same reasons as described in Section 2.4.1.

### 2.5. Instruments

#### 2.5.1. OSCE Checklist

The OSCE checklist was developed based on a literature search. The original OSCE checklist encompassed 39 items. For subsequent analysis, two items of the checklist had to be excluded because two aspects could not be performed by every standardized patient or in every OSCE encounter, respectively. Consequently, a maximum of 37 points was achievable, which were divided into the sections “general preparation of blood pressure measurement,” “resting phase,” “steps of BP measurement,” and “documentation”. Supplemental Material S1 summarizes the content of the checklist. Every item was weighted equally. If an item was fulfilled, 1 point was awarded, whereas 0 points were awarded if the participant did not comply with an item. The OSCE score was used to assess the BP measurement performance and consequently to evaluate the acquisition of skill by the respective instruction.

#### 2.5.2. Self-Assessment Survey

To evaluate the impact of the two instruction methods on the participants’ self-assessment of their proficiency and confidence in their BP measurement skills, the participants completed a survey (Supplementary Material S2) immediately before each OSCE session. The self-assessment survey encompassed five items. A six-point Likert scale (0 = strongly disagree, 1 = disagree, 2 = rather disagree, 3 = rather agree, 4 = agree, 5 = strongly agree) was used. The participants’ demographics, such as age, gender, additional education as a pharmaceutical technician assistant, current or former work in a community pharmacy and former experience in BP measurement, and preparation for BP measurement, were collected in a questionnaire along with the self-assessment survey.

#### 2.5.3. Perception and Satisfaction Survey

The participants were asked to fill out a perception and satisfaction survey about OSCEs, the respective instructional approach, and the entire OSCE seminar. The survey consisted of 13 items rated by a six-point Likert scale from “strongly disagree” to “strongly agree”. One item was solely applicable to the SIV group and another item was solely applicable to the FTFI group. Additionally, for each group, one question was included where the participants were required to rate the instructional approach they received, based on the German school grading scale, ranging from 1 = very good to 6 = inadequate. In free-text items, they also were asked about what they favored most and what they would suggest changing. For analysis, the comments on the free-text items were categorized into topics.

### 2.6. Statistical Analysis

All data, except for the perception and satisfaction survey, were collected in a de-identified way, and rendered anonymous after data analysis. The perception and satisfaction survey was collected anonymously. The participants were randomized to the SIV group and FTFI group using R [35]. The non-inferiority of the SIV compared to the FTFI was examined with a two-sided 95% confidence interval (CI) for the difference in the mean change of OSCE score between the two groups in terms of the primary outcome, and for the difference in the mean change of self-assessment score between the two groups in terms of the secondary outcome. The difference in the mean change of the respective score between the two groups was calculated by: “mean change of the respective score of the SIV group” minus “mean change of the respective score of the FTFI group”. Thereby, the mean change of the respective score for each group was determined by calculating the post-instruction score minus the pre-instruction score for each included participant and subsequently calculating the mean.

We defined a non-inferiority margin of −10% to be educationally meaningful. This decision, made before collecting the data, was based on a non-inferiority study by Platz et al., who compared different educational methods in medicine and surgery residents [36]. In our study, −10% corresponds to −3.7 points regarding the OSCE score and −2.5 points regarding the self-assessment score. If the two-sided 95% confidence interval lay entirely to the right of the non-inferiority margin, non-inferiority for the respective objective was claimed. If the condition of non-inferiority was fulfilled for the respective score, we assessed whether the increase in the respective score was higher in the SIV group than in the FTFI group. This was tested using a one-sided Mann–Whitney test with a significance level of alpha = 0.05.

The change in the OSCE score and self-assessment score from pre- to post-instruction assessment for each group was analyzed using a one-sided paired two-sample Wilcoxon signed rank test with a significance level of alpha = 0.05. The difference between the two groups in the baseline (pre-instruction assessment) scores was analyzed using a two-sided Mann–Whitney test with a significance level of alpha = 0.05. In the perception and satisfaction survey, participants’ ratings of the respective instruction were analyzed using a two-sided Mann–Whitney test with a significance level of alpha = 0.05. Asymptotic *p*-values are stated. The *p*-values were not adjusted for multiple testing. Microsoft Excel [37] was used for data entry, and Microsoft Excel and OriginPro [38] were used for analysis.

## 3. Results

Of 58 students who were in their last semester, 10 did not provide informed consent and two were trained as raters before the participant recruitment. Consequently, 46 students participated in the study. A total of 23 participants were randomized to the FTFI group, and 23 participants to the SIV group. All 46 participants completed the pre- and post-instruction assessment. One participant of the FTFI group was excluded from the analysis due to the standardized patient’s non-compliance with the predefined setting, and one participant of the SIV group was excluded from the analysis because of incomplete self-assessment survey data. Consequently, 22 participants of the FTFI group and 22 participants of the SIV group were included in the analysis. The two excluded participants could not be excluded from the analysis of the perception and satisfaction survey due to its anonymous form. Forty-one participants took part in the perception and satisfaction survey.

Out of 21 participants of the FTFI group who filled in the items regarding demographics, 71.43% were female, 9.52% had training as a pharmaceutical technical assistant, and 28.57% had measured the BP with an oscillometric device for the first time. Out of 22 participants of the SIV group who filled in the items regarding demographics, 72.73% were female, 13.64% had training as a pharmaceutical technical assistant, and 45.45% had measured the BP with an oscillometric device for the first time. As one demographic item regarding experience in BP measurement had been worded ambiguously, it was excluded from the analysis. Appendix A provides further details regarding the demographics.

### 3.1. OSCE Score

At baseline (pre-instruction assessment), the OSCE score did not differ significantly between the two groups (*p* = 0.620). Both the FTFI group (*p* < 0.001) and SIV group (*p* < 0.001) demonstrated a significant improvement in the OSCE score from pre- to post-instruction assessment after the respective instruction. The detailed results for the OSCE score are depicted in Table 1.

**Figure 2:**
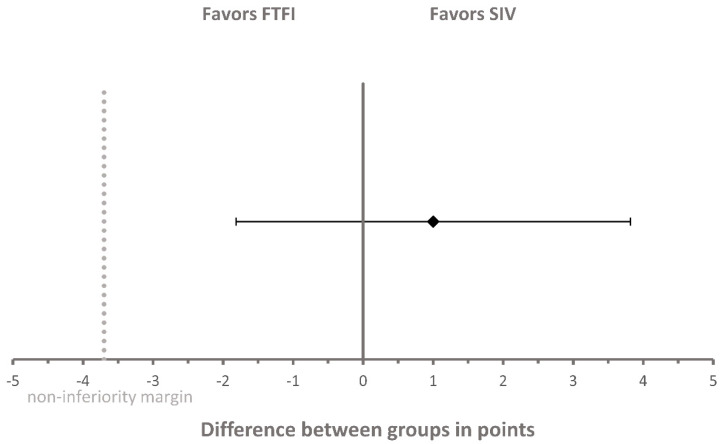
Non-inferiority analysis of OSCE score between the SIV group and FTFI group. The diamond represents the difference in mean change of OSCE score between the two groups and was calculated as follows: “mean change of OSCE score of the SIV group” minus “mean change of OSCE score of the FTFI group”. Error bars indicate the two-sided 95% confidence interval. The dashed line represents the non-inferiority margin of −3.7 points. OSCE = objective structured clinical examination; FTFI = face-to-face instruction; SIV = self-instruction video.

Regarding the non-inferiority analysis of the primary objective, there was a one-point difference in the mean change of OSCE score between the groups, with a 95% confidence interval (CI) of −1.82 points to 3.82 points (Figure 2). The two-sided 95% CI of the difference in the mean change in OSCE score between the two groups lay entirely to the right of the non-inferiority margin of −3.7 points (≙ −10%). Therefore, we concluded that the SIV is non-inferior to FTFI for acquiring BP measurement skills (primary outcome). When comparing the changes in OSCE scores (from pre- to post-instruction OSCE) between the two groups, the OSCE score in the SIV group did not show a significantly greater increase compared to the FTFI group (*p* = 0.262).

### 3.2. Self-Assessment Score

At baseline (pre-instruction assessment), the self-assessment score (*p* = 0.407) did not differ significantly between the two groups. In both the FTFI group (*p* < 0.001) and SIV group (*p* = 0.001), the self-assessment score representing the participants’ self-confidence showed a significant improvement from pre- to post-instruction assessment. The detailed results of the self-assessment score are depicted in Table 1.

**Figure 3:**
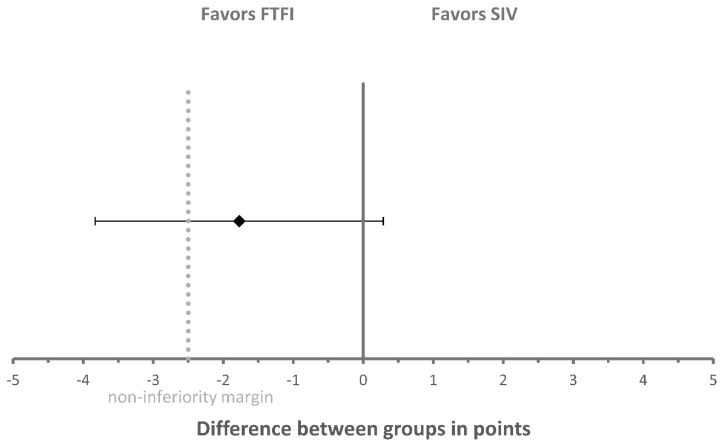
Non-inferiority analysis of self-assessment score between the SIV group and FTFI group. The diamond represents the difference in mean change of self-assessment score between the two groups and was calculated as follows: “mean change of self-assessment score of the SIV group” minus “mean change of self-assessment score of the FTFI group”. Error bars indicate the two-sided 95% confidence interval. The dashed line represents the non-inferiority margin of −2.5 points. FTFI = face-to-face instruction; SIV = self-instruction video.

Regarding the non-inferiority analysis of the secondary outcome of the self-assessment score, the difference in the mean change of self-assessment score between the two groups was −1.77 points, with a 95% confidence interval (CI) of −3.83 points to 0.29 points (Figure 3). Thus, the 95% CI of the difference in the mean change in self-assessment score between the two groups included the non-inferiority margin of −2.5 points (≙ −10%) and, consequently, the result regarding non-inferiority was inconclusive.

### 3.3. Perception and Satisfaction Survey

Out of 46 randomized participants, 41 (20 from the FTFI group, 21 from the SIV group) took part in the perception and satisfaction survey. However, not all participants filled out the survey entirely. Therefore, the total number of responses differed depending on the item. Only the items filled unambiguously were included in the evaluation. The results regarding the items are depicted in Appendix B. FTFI was rated with a mean German school grade of 2.32 (n = 19, standard deviation (SD): 0.89) and a median of 2 (n = 19, first quartile: 2, third quartile: 3). The SIV was awarded a mean German school grade of 1.71 (n = 21, standard deviation (SD): 0.64) and a median of 2 (n = 21, first quartile: 1, third quartile: 2); the grades between the two groups differed significantly (*p* = 0.026). This result indicates that the SIV was more appreciated than the FTFI. Regarding the free-text items, the three most frequent topics of each item per group are shown in Appendix C.

## 4. Discussion

In this randomized controlled study, both educational approaches, i.e., FTFI and SIV, were aimed at conveying the necessary knowledge and skills for performing correct and accurate BP measurements. In this study, the SIV was non-inferior to FTFI in the acquisition of BP measurement skills in pharmacy students (primary outcome). Furthermore, the participants’ self-confidence improved. However, the analysis of self-confidence yielded an inconclusive result regarding the non-inferiority (secondary outcome). Finally, the students’ perception and satisfaction (secondary outcome) indicated that the seminar as a whole, the integrated OSCEs, and both instruction approaches were predominately well received.

Our findings imply that, as a replacement for instructor-led teaching on BP measurement skills, an SIV achieves a comparable effect on students’ performance compared to FTFI. This corresponds to the findings of George et al., who also demonstrated non-inferiority of video demonstration as a replacement for bedside teaching in medical students. In line with our study, they applied a non-inferiority margin of 10% [32]. The setting of an SIV can influence the results of such investigations. De Vries et al., for example, revealed that self-directed video-based training with shorter duration compared to instructor-led training was insufficient to achieve comparable results in laypeople [39]. In our study and that of George et al. [32], the video-based instruction had rather a similar duration compared to the traditional instructor-led instruction. Furthermore, it should be considered that De Vries et al. [39] showed the video to laypeople, while in our study and that of George et al. [32], the video was viewed by health professions students.

Although there was improved participants’ self-confidence in both groups in our study, our findings on the participants’ self-confidence were inconclusive regarding non-inferiority. Lee et al. found that the frequency of viewing an instructional video was correlated with confidence in practice [40]. As the participants of the SIV group had a limited 15-min time frame of access to the video, it might be speculated that the limited video access provided in our study setting was insufficient for building up enough confidence. We can only assume that unlimited video access with more frequent viewing of the instructional video would have resulted in a more strengthened data set regarding self-confidence so that non-inferiority could have been statistically demonstrated.

The instructional video on BP measurement was highly accepted by the students. In particular, the responses of both groups implied that the students appreciated the implementation of instructional videos in clinical pharmacy education. Interestingly, the literature indicates that students are critical of instructional videos as a standalone approach [32,41]. For example, Bazyk et al. reported that, although students considered the learning experience with the video as good, they preferred live instruction over videotaped instruction. They appreciated the interaction with the instructor and the possibility of asking questions immediately in case of uncertainty, during live instruction [41]. Lwin et al., however, reported high overall satisfaction among participants with self-directed interactive video-based instruction, whereby satisfaction was surveyed only among participants of the video group [19].

In our study, although both instructional approaches led to a significant increase in the participants’ BP measurement skills and self-confidence, there remains potential for further improvement in both groups. We assume that enhanced skill performance and confidence might be achieved by a more extensive teaching approach than that applied in our study. For example, combining the respective instructional approach with other teaching activities such as practice time and instructor feedback may be more beneficial to performance and self-confidence. Neither group had practice time. However, the pre-instruction OSCE in our study may have induced a learning effect [42]. In general, depending on the skill intended to be taught, a combination of an instructional video with instructor-led teaching may be necessary.

Finally, in our study, the conveying of BP measurement skills was investigated. The next step is to counsel and advise the patient appropriately regarding their obtained BP. We suggest that future research on BP measurement skills should extend the investigation on this step.

### Limitations

There are some limitations to our study. First, we mitigated the potential inter- and intra-rater variability by training the raters beforehand. Moreover, the FTFI was performed by three different instructors, which may have led to inconsistency in the information and techniques taught [43]. To reduce the inter-instructor variability, the lead instructor trained the two other instructors, and a script for the FTFI was provided. Another aspect that should be considered is that the pre- and post-instruction OSCEs were the same OSCE case. This was done because we aimed to create a standardized study environment with comparable pre- and post-instruction assessment. To minimize the possibility that participants across the groups shared information, the participants were instructed verbally and via a confidentiality agreement not to disclose information on the instruction method and the OSCEs. To reduce the possibility of information on the SIV being shared, we did not make the video available online. Additionally, we set the intervention of the groups on the same day and same timeframe to reduce cross-communication between the groups.

## 5. Conclusions

Our study demonstrates that, based on the OSCE score and a non-inferiority margin of −10%, the SIV was non-inferior to FTFI for acquiring BP measurement skills with an oscillometric device in pharmacy. As for the results of the self-assessment score, the result was inconclusive regarding non-inferiority. Considering the advantages of instructional videos and our findings, SIVs might be considered as a valuable option for teaching BP measurement skills. However, depending on the skill intended to be taught, a combination of an instructional video with instructor-led teaching may be necessary.

## Figures and Tables

**Table 1:** OSCE score and self-assessment score by group.

Group	Instrument	Pre-Instruction Assessment Score in Points		Post-Instruction Assessment Score in Points		Change in Points		*p*-Value
		Mean (SD)	Median (IQR)	Mean (SD)	Median (IQR)	Mean (SD)	Median (IQR)	
**FTFI**	OSCE checklist	7.95	8	20.32	20	12.36	11.5	*p* < 0.001
	(n = 22)	(3.91)	(4)	(3.20)	(4)	(4.51)	(7)	
	Self-assessment survey	12.91	14	18.05	18	5.14	4.5	*p* < 0.001
	(n = 22)	(3.48)	(4)	(2.66)	(2)	(2.62)	(4)	
**SIV**	OSCE checklist	7.41	6	20.77	20.5	13.36	13.5	*p* < 0.001
	(n = 22)	(3.85)	(7)	(4.28)	(5)	(5.01)	(8)	
	Self-assessment survey	14.14	14.5	17.5	17.5	3.36	3	*p* = 0.001
	(n = 22)	(4.38)	(7)	(2.43)	(3)	(4.17)	(7)	

OSCE = objective structured clinical examination; FTFI = face-to-face instruction; SIV = self-instruction video; SD = standard deviation; IQR = interquartile range; The *p*-value was calculated by one-sided paired two-sample Wilcoxon signed rank test. A significance level of alpha = 0.05 was used.

## Supplementary Materials

The following are available online at https://www.mdpi.com/2226-4787/8/4/217/s1, Material S1: Summarized content of the OSCE checklist of the blood pressure measurement, Material S2: Self-assessment survey.

## Appendix A

**Table A1:** Demographics of participants.

	Face-to-Face Instruction Group (FTFI Group) (n = 21 ^1^)	Self-Instruction Video Group (SIV Group) (n = 22 ^2^)
**Age in years**		
Median (IQR)	22 (3)	24.5 (6)
Mean (SD)	23.86 (3.17)	27.14 (7.91)
**Gender**		
Female, n (%)	15 (71.43)	16 (72.73)
Male, n (%)	6 (28.57)	6 (27.27)
**Training as a pharmaceutical technical assistant**		
Yes, n (%)	2 (9.52)	3 (13.64)
No, n (%)	19 (90.48)	19 (86.36)
**Currently or formerly worked in a community pharmacy**		
Yes, n (%)	6 (28.57)	3 (13.64)
No, n (%)	15 (71.43)	19 (86.36)
**Have measured BP with an oscillometric device for the first time**		
Yes, n (%)	6 (28.57)	10 (45.45)
No, n (%)	15 (71.43)	12 (54.55)
**Preparation for BP measurement task at pre-instruction assessment**		
Yes, n (%)	0 (0)	1 (4.55)
No, n (%)	21 (100)	21 (95.45)
**Preparation for BP measurement task at post-instruction assessment ^2^**		
Yes, n (%)	0 (0)	3 (13.64)
No, n (%)	22 (100)	19 (86.36)

^1^ One participant of the FTFI group did not fill in the demographic section in the self-assessment survey at pre-instruction assessment. ^2^ At post-instruction assessment, 22 participants of the FTFI group filled in the demographic section. IQR = interquartile range; SD = standard deviation; BP = blood pressure.

## Appendix B

**Table A2:** Results of the perception and satisfaction survey.

	Proportion of Responses, n (%)					
	Strongly Disagree	Disagree	Rather Disagree	Rather Agree	Agree	Strongly Agree
1. I enjoyed the OSCE seminar on BP measurement.						
FTFI group; n = 20	0 (0)	2 (10)	2 (10)	7 (35)	8 (40)	1 (5)
SIV group; n = 21	0 (0)	1 (4.76)	3 (14.29)	4 (19.05)	12 (57.14)	1 (4.76)
2. During the OSCEs/simulations, I was able to determine my strengths and weaknesses on BP measurement.						
FTFI group; n = 20	0 (0)	0 (0)	0 (0)	2 (10)	8 (40)	10 (50)
SIV group; n = 21	0 (0)	1 (4.76)	0 (0)	6 (28.57)	9 (42.86)	5 (23.81)
3. The level of difficulty of the OSCE case was appropriate.						
FTFI group; n = 20	0 (0)	0 (0)	0 (0)	4 (20)	7 (35)	9 (45)
SIV group; n = 20	0 (0)	1 (5)	1 (5)	1 (5)	9 (45)	8 (40)
4. Ten minutes was an appropriate timeframe to complete the OSCE case.						
FTFI group; n = 20	0 (0)	1 (5)	1 (5)	4 (20)	6 (30)	8 (40)
SIV group; n = 21	1 (4.76)	1 (4.76)	1 (4.76)	2 (9.52)	10 (47.62)	6 (28.57)
5. The instruction (FTFI or SIV) should take place on a different day than the OSCEs/simulations.						
FTFI group; n = 20	4 (20)	6 (30)	1 (5)	4 (20)	3 (15)	2 (10)
SIV group; n = 20	4 (20)	10 (50)	2 (10)	0 (0)	2 (10)	2 (10)
6. The OSCEs/simulations enabled me to apply the knowledge and skills that I acquired during the instruction.						
FTFI group; n = 20	0 (0)	0 (0)	0 (0)	3 (15)	10 (50)	7 (35)
SIV group; n = 21	0 (0)	0 (0)	1 (4.76)	6 (28.57)	9 (42.86)	5 (23.81)
7. After this seminar, I feel better prepared for the correct BP measurement in the community pharmacy.						
FTFI group; n = 20	0 (0)	0 (0)	0 (0)	4 (20)	7 (35)	9 (45)
SIV group; n = 21	0 (0)	0 (0)	0 (0)	4 (19.05)	12 (57.14)	5 (23.81)
8. Carrying out the OSCEs/simulations increased my self-confidence in performing BP measurements on real patients in the community pharmacy.						
FTFI group; n = 20	0 (0)	0 (0)	2 (10)	6 (30)	5 (25)	7 (35)
SIV group; n = 21	0 (0)	0 (0)	0 (0)	4 (19.05)	13 (61.90)	4 (19.05)
9. The SIV was helpful for the preparation for the OSCEs/simulations.						
FTFI group; n = 0	Not applicable					
SIV group; n = 21	0 (0)	0 (0)	2 (9.52)	3 (14.29)	7 (33.33)	9 (42.86)
10. The FTFI was helpful for the preparation for the OSCEs/simulations.						
FTFI group; n = 20	0 (0)	0 (0)	2 (10)	5 (25)	7 (35)	6 (30)
SIV group; n = 0	Not applicable					
11. In the future, instruction videos should be included in the teaching of clinical pharmacy.						
FTFI group; n = 11	0 (0)	0 (0)	0 (0)	2 (18.18)	4 (36.36)	5 (45.45)
SIV group; n = 20	0 (0)	0 (0)	2 (10)	4 (20)	7 (35)	7 (35)
12. OSCEs/simulations about BP measurement are superfluous because one can do nothing wrong with the BP measurement.						
FTFI group; n = 18	11 (61.11)	7 (38.89)	0 (0)	0 (0)	0 (0)	0 (0)
SIV group; n = 20	14 (70)	5 (25)	1 (5)	0 (0)	0 (0)	0 (0)
13. In the future, OSCEs/simulations for training of practical skills (such as BP measurement) should be included as a regular part of the clinical pharmacy course.						
FTFI group; n = 16	0 (0)	0 (0)	0 (0)	3 (18.75)	9 (56.25)	4 (25)
SIV group; n = 21	0 (0)	1 (4.76)	1 (4.76)	3 (14.29)	8 (38.10)	8 (38.10)

BP = blood pressure, OSCE = objective structured clinical examination; FTFI = face-to-face instruction; SIV = self-instruction video.

## Appendix C

**Table A3:** Exemplary topics of comments on free-text items of the perception and satisfaction survey.

Group	Free-Text Item	Topics
**Face-to-face instruction group**	What did you particularly like about the face-to-face instruction?	“the possibility to ask questions”
		“demonstration of procedure”
		“the detailed instruction”
	I would change the following about the face-to-face instruction:	“the fast pace of speech or demonstration”
		“information should be put into writing (e.g., handout, slides, or flipchart)”
		“the duration of the instruction was too short”
**Self-instruction video group**	What did you particularly like about the self-instruction video?	“the combination of text and video”
		“the actors”
		“the descriptive explanation”
	I would change the following about self-instruction video:	“narrative voice for the written slides should be implemented”
		“taking notes should be allowed”
		“summary in bullet point form should be added to the end of the video”

The three most frequent topics of comments are presented for each item per group. If topics appeared with equal frequency, one topic was chosen.
