# Supplementary material for: Self-Instruction Video Versus Face-to-Face Instruction of Pharmacy Students’ Skills in Blood Pressure Measurement
Source: Pharmacy (Basel). 2020 Nov 16;8(4):217. doi: 10.3390/pharmacy8040217 (PMC7711835; doi:10.3390/pharmacy8040217)
Supplement: Supplementary file 1 [file pharmacy-08-00217-s001.zip › supplemental mateial 1.pdf]

Supplemental Material 1: Summarized content of the OSCE checklist of the blood pressure measurement

Section	Number of Items in the Respective Section	Summarized Content
General Preparation of Blood Pressure Measurement	9	• Examinee asks questions about factors that potentially could affect BP (6 items)<br>• Examinee inquires whether measuring arm is known (1 item)<br>• Examinee asks the patient to remove clothing from arm (or arms respectively, if both arms are intended to be measured) without rolling up sleeves (1 item)<br>• Examinee measures arm circumference/s (1 item)
Resting Phase	4	• Examinee gives instructions to the patient regarding the patient's position during the rest period (2 items)<br>• Examinee gives instructions to the patient regarding the patient's attitude during the rest period (1 item)<br>• Examinee lets the patient relax (rest) for 5 minutes in a seated position (before measurement) (1 item)
Steps of the Blood Pressure Measurement	18	• Examinee selects correct cuff size (1 item)<br>• Examinee places the cuff correctly (5 items)<br>• Examinee gives instructions to the patient regarding the patient's body position, arm position, and attitude during BP measurement (5 items)<br>• Examinee measures BP correctly (5 items)<br>• Examinee reports the measured BP appropriately to the patient and asks for open questions (2 items)
Documentation	6	• Examinee documents properly (6 items)

BP= blood pressure
