# Supplementary material for: Self-Instruction Video Versus Face-to-Face Instruction of Pharmacy Students’ Skills in Blood Pressure Measurement
Source: Pharmacy (Basel). 2020 Nov 16;8(4):217. doi: 10.3390/pharmacy8040217 (PMC7711835; doi:10.3390/pharmacy8040217)
Supplement: Supplementary file 1 [file pharmacy-08-00217-s001.zip › supplemental material 2.pdf]

Self-assessment survey

Coding of participant: ______________________________

Date: ____

How do you rate your competence in the following areas?

Please tick the boxes.

---

1. I feel able to correctly measure blood pressure in the community pharmacy.	☐ Strongly disagree	☐ Disagree	☐ Rather disagree	☐ Rather agree	☐ Agree	☐ Strongly agree
2. I feel confident in the use of oscillometric upper-arm blood pressure monitors.	☐ Strongly disagree	☐ Disagree	☐ Rather disagree	☐ Rather agree	☐ Agree	☐ Strongly agree
3. I feel confident in choosing the proper blood pressure monitor.	☐ Strongly disagree	☐ Disagree	☐ Rather disagree	☐ Rather agree	☐ Agree	☐ Strongly agree
4. I know the necessary steps for an accurate blood pressure measurement with an oscillometric upper-arm blood pressure monitor.	☐ Strongly disagree	☐ Disagree	☐ Rather disagree	☐ Rather agree	☐ Agree	☐ Strongly agree
5. If hypothetically, I am given the task of measuring blood pressure again in a month, I will be able to measure blood pressure accurately.	☐ Strongly disagree	☐ Disagree	☐ Rather disagree	☐ Rather agree	☐ Agree	☐ Strongly agree
